# Rapidly declining skeletal muscle mass predicts poor prognosis of hepatocellular carcinoma treated with transcatheter intra-arterial therapies

**DOI:** 10.1186/s12885-018-4673-2

**Published:** 2018-07-24

**Authors:** Takamasa Kobayashi, Hirokazu Kawai, Oki Nakano, Satoshi Abe, Hiroteru Kamimura, Akira Sakamaki, Kenya Kamimura, Atsunori Tsuchiya, Masaaki Takamura, Satoshi Yamagiwa, Shuji Terai

**Affiliations:** Division of Gastroenterology and Hepatology, Niigata University Graduate School of Medical and Dental Sciences, 1-757 Asahimachi-dori Chuo-ku, Niigata, 951-8510 Japan

**Keywords:** Skeletal muscle loss, Sarcopenia, Hepatocellular carcinoma, Transcatheter arterial chemoembolization, Transcatheter arterial infusion chemotherapy, Prognosis

## Abstract

**Background:**

The impact of sarcopenia on the prognosis of patients with hepatocellular carcinoma (HCC) who receive transcatheter intra-arterial therapies, including transcatheter arterial chemoembolization and transcatheter arterial infusion chemotherapy, remains unclear. We investigated the prognostic value of skeletal muscle loss (SML) stratified by cutoffs for sarcopenia and rate of change in skeletal muscle mass over 6 months.

**Methods:**

We retrospectively evaluated 102 patients with HCC treated with transcatheter intra-arterial therapies between 2005 and 2015. Computed tomography images of the third lumbar vertebra (L3) were analyzed to obtain the skeletal muscle area normalized for the height squared, defined as the skeletal muscle index at L3 (L3 SMI), before and 6 months after treatment. Low or high SMI was defined using cutoff values of 42 cm^2^/m^2^ in men and 38 cm^2^/m^2^ in women. The rate of change in skeletal muscle mass (ΔL3 SMI) over 6 months was calculated. Overall survival (OS) was compared in groups classified by baseline L3 SMI and ΔL3 SMI; prognostic significance was assessed with univariate and multivariate analyses, using Cox proportional hazards models.

**Results:**

OS did not differ significantly between groups with low (*n* = 31) and high (*n* = 71) SMI at baseline (*P* = 0.172), but OS was significantly poorer in patients with SML (*n* = 41), defined as ΔL3 SMI < − 4.6% over 6 months than in those without SML (*n* = 61, *P* = 0.018). On multivariate analysis, SML (hazard ratio [HR], 1.675; 95% confidence interval [CI], 1.031–2.721; *P* = 0.037), serum alpha-fetoprotein ≥20 ng/mL (HR, 2.550; 95% CI, 1.440–4.515; *P* = 0.001), and maximum tumor diameter ≥ 30 mm (HR, 1.925; 95% CI, 1.166–3.179; *P* = 0.010) were independent predictors of poor OS. Baseline L3 SMI was not significantly associated with OS (HR, 1.405; 95% CI, 0.861–2.293; *P* = 0.174).

**Conclusions:**

ΔL3 SMI was an independent prognostic factor in patients with HCC treated with transcatheter intra-arterial therapies. Further study is required to reveal whether prevention of skeletal muscle depletion might be a new therapeutic strategy to contribute to improved clinical outcomes in patients with HCC.

## Background

Hepatocellular carcinoma (HCC) is one of the most common malignancies worldwide and a major cause of cancer mortality [1]. Recent advances in therapeutic modalities, including hepatic resection, percutaneous ethanol injection, radiofrequency ablation (RFA), and transplantation, have substantially improved the prognosis of patients with HCC [2–6]. Many patients with advanced HCC or severely impaired liver function, however, are beyond the indications for these radical treatments. Transcatheter intra-arterial therapies, including transcatheter arterial chemoembolization (TACE) and transcatheter arterial infusion chemotherapy (TAI), are widely used in advanced HCC. Although these treatments are not curative and yield a lower cumulative survival rate than surgical resection and local ablation therapies [7], the prognosis of patients receiving these interventions fluctuates according to various factors, such as hepatic functional reserve; tumor number, diameter, and distribution in the liver; and treatment efficacy [8–11].

Sarcopenia is a geriatric syndrome characterized by progressive and generalized loss of skeletal muscle mass and strength. The etiology of sarcopenia is multifactorial, comprising primary causes associated with age and secondary causes related to reduced physical activity, diseases, and nutritional disorders [12, 13]. Several recent studies have reported close associations between sarcopenia and poor clinical outcomes in patients with various types and stages of malignancies, including HCC [14, 15]. Furthermore, loss of skeletal muscle mass has been associated with poorer overall survival (OS) and disease-free survival in patients with HCC treated with surgical resection or RFA [16–20]. However, the impact of sarcopenia on the prognosis of patients with HCC who receive transcatheter intra-arterial therapies has not yet been assessed.

We hypothesized that sarcopenia may affect the clinical outcomes of patients with HCC who experience gradual deterioration of hepatic function and nutritional status during repeated rounds of transcatheter intra-arterial therapy. Therefore, we retrospectively investigated the prognostic significance of loss of skeletal muscle mass in a group of these patients, stratified by cutoff values for sarcopenia [21]. We also evaluated the prognostic value of the rate of change in skeletal muscle mass over 6 months in this group.

## Methods

### Patients

In the present study, we retrospectively analyzed patients with HCC who underwent TACE and/or TAI as initial treatment at Niigata University Medical and Dental Hospital between January 2005 and December 2015. HCC was diagnosed principally based on its enhancement pattern on dynamic computed tomography (CT) or dynamic magnetic resonance imaging (MRI), exhibiting contrast enhancement in the arterial phase followed by rapid washout in the portal or equilibrium phase. Patients who had undergone a CT examination within 30 days prior to treatment and about 6 months (range, 90–270 days) after treatment were included in the study, while patients who received treatment either initially or additionally with curative methods, including surgical resection or RFA, were excluded.

This retrospective study was approved by the ethics committee of the Niigata University School of Medicine (approval number 2442) and was conducted in accordance with the 1975 Helsinki Declaration. Because of the anonymous nature of the data, the requirement for additional informed consent to participate in the study was waived.

### Treatment procedure

TACE and/or TAI were performed according to the clinical practice guidelines for HCC of the Japan Society of Hepatology [22]. Briefly, TACE or TAI is recommended for patients with multiple tumors and Child–Pugh class A or B hepatic impairment severity. The TAI procedure consists of the injection of cisplatin (IA-call®; Nippon Kayaku Co., Ltd., Tokyo, Japan), miriplatin (MIRIPLA®; Dainippon Sumitomo Pharma Co., Ltd., Osaka, Japan), or an emulsion of epirubicin (Farmorubicin®; Pfizer Japan Inc., Tokyo, Japan) in lipiodol into hepatic arteries, including tumor-nourishing arteries. TACE includes subsequent embolization of the feeding arteries with gelatin sponge particles (Gelpart®; Nihonkayaku, Tokyo, Japan) following the TAI procedure. TAI without embolization was implemented when multiple tumors were extensively distributed bilaterally in the lobes of the liver, the arterial anatomy precluded a super-selective injection, or there were significant arteriovenous fistulas or tumor thrombi in the main trunk of the portal vein.

### Skeletal muscle mass measurement

Skeletal muscle areas were measured on axial sections at the level of the third lumbar vertebra (L3) of pre- and post-treatment CT images, using SliceOmatic software (version 5.0; TomoVision Inc., Montreal, Canada). The psoas, erector spinae, quadratus lumborum, transversus abdominis, external and internal oblique, and rectus abdominis muscles were delineated by density thresholds ranging from − 29 to 150 Hounsfield units [23]. The muscle area (cm^2^) at the L3 level was normalized by the square of height (m^2^) to obtain the skeletal muscle index at L3 (L3 SMI, cm^2^/m^2^). Patients were classified into low or high SMI groups based on cutoff values of 42 cm^2^/m^2^ in men and 38 cm^2^/m^2^ in women, values recommended in the guidelines for sarcopenia in liver disease described by the Japan Society of Hepatology [21].

The rate of change in skeletal muscle mass over 6 months was calculated using the following formula: rate of change in skeletal muscle mass over 6 months (ΔL3 SMI, %) = (L3 SMI on post-treatment CT − L3 SMI on pre-treatment CT)/(L3 SMI on pre-treatment CT)/(days between pre- and post-treatment CT examinations) × 180 × 100. The cutoff value of ΔL3 SMI was determined by optimal stratification of survival based on the most significant *P* value in log-rank statistics [24, 25].

### Clinical data

Retrospectively collected demographic and clinical data included age, sex, and body mass index (BMI); hepatitis B virus (HBV) surface antigen; anti-hepatitis C virus (HCV) antibody; serum concentrations of alanine aminotransferase (ALT), total bilirubin, albumin, and alpha-fetoprotein (AFP); platelet count; Child–Pugh classification; TNM stage according to the Liver Cancer Study Group of Japan [26]; maximum tumor diameter; number of tumors; presence or absence of branched-chain amino acid (BCAA) supplementation; number of sessions of transcatheter intra-arterial therapy between the pre- and post-treatment CT examinations; transcatheter intra-arterial treatment modality; treatment response evaluated according to the Modified Response Evaluation Criteria in Solid Tumors (mRECIST) [27]; and cause of death.

### Statistical analysis

Continuous variables expressed as median and range were compared using Mann–Whitney *U* tests, and categorical variables expressed as number and percentage were compared using Fisher’s exact tests or chi-squared tests. Survival days were counted from the date of pre-treatment CT. The Kaplan-Meier method was used to estimate survival rates, which were then compared using log-rank tests. The prognostic significance of covariates was assessed with univariate and multivariate analyses using a Cox proportional hazards model and expressed as hazard ratios (HRs) and 95% confidence intervals (CIs). All variables significant in univariate analyses were entered into multivariate models. All statistical analyses were performed using IBM SPSS Version 21 Statistics software (IBM Corp., Armonk, NY, USA). All tests were two-sided, and *P* values < 0.05 were considered statistically significant.

## Results

### Baseline demographic and clinical characteristics of patients

Of the 621 consecutive patients admitted to our hospital for HCC treatment between January 2005 and December 2015, 102 were deemed eligible for participation in the current study; the baseline demographic and clinical characteristics of these patients are shown in Table 1. The median observation period was 733 days (range, 153–3278 days). The median age was 69 years (range, 34–89 years), and 70 patients (68.6%) were men. The median L3 SMI on pre-treatment CT in men and women was 47.1 cm^2^/m^2^ (range, 31.5–64.4 cm^2^/m^2^) and 36.5 cm^2^/m^2^ (range, 25.6–55.6 cm^2^/m^2^), respectively. Fifty-nine patients (57.8%) received a combination of TACE and TAI. A total of 167 treatment sessions were administered; among these, adverse events occurred in nine sessions, including prolonged fever in six sessions and liver function impairment in three sessions. No treatment-related deaths occurred. Seventy-two patients (70.6%) died during the study period, with liver-related death (57 patients, 79.2%) the leading cause of mortality.

**Table 1:** Baseline demographic and clinical characteristics

Characteristics	Value
Number of patients	102
Observation period, days, median [range]	733 [153–3278]
Age, years, median [range]	69 [34–89]
Sex (men), *n* (%)	70 (68.6)
BMI in men, kg/m^2^, median [range]	23.5 [16.2–33.8]
BMI in women, kg/m^2^, median [range]	23.0 [16.0–37.5]
Baseline L3 SMI in men, cm^2^/m^2^, median [range]	47.1 [31.5–64.4]
Baseline L3 SMI in women, cm^2^/m^2^, median [range]	36.5 [25.6–55.6]
Etiology, *n* (%)	
HBV	11 (10.8)
HCV	50 (49.0)
NBNC	41 (40.2)
Alcohol	26 (25.5)
NASH	7 (6.9)
PBC	2 (2.0)
Cryptogenic	6 (5.9)
ALT, U/L, median [range]	37 [9–243]
Total bilirubin, mg/dL, median [range]	1.0 [0.4–3.5]
Albumin, g/dL, median [range]	3.7 [2.0–4.9]
Platelet count, × 10^4^/μL, median [range]	12.1 [2.2–80.9]
Child-Pugh classification (A/B/C), *n* (%)	68(66.7)/34(33.3)/0(0)
AFP, ng/mL, median [range]	21.8 [1.0–633,900]
TNM stage (I/II/III/IV), *n* (%)	11(10.8)/22(21.6)/46(45.1)/23(22.5)
Maximum tumor diameter, mm, median [range]	32 [9–198]
Number of tumors (solitary/multiple), *n* (%)	29(28.4)/73(71.6)
Treatment sessions between CT exams, sessions, median [range]	1 (1–6)
Treatment modality (TACE/TAI/TACE+TAI), *n* (%)	18(17.6)/25(24.5)/59(57.8)
mRECIST assessment (CR/PR/SD/PD), *n* (%)	18(17.6)/26(25.5)/9(8.8)/49(48.0)
BCAA supplementation (presence/absence), *n* (%)	72(70.6)/30(29.4)
Cause of death (liver-related/infection/GI bleeding/others/unknown), *n*	57/7/2/4/2

*BMI* body mass index, *L3 SMI* skeletal muscle index at third lumber vertebral level, *HBV* hepatitis B virus, *HCV* hepatitis C virus, *NBNC* non-B non-C, *NASH* non-alcoholic steatohepatitis, *PBC* primary biliary cholangitis, *ALT* alanine aminotransferase, *AFP* alpha-fetoprotein, *TACE* transcatheter arterial chemoembolization, *TAI* transcatheter arterial infusion chemotherapy, *mRECIST* Modified Response Evaluation Criteria in Solid Tumors, *CR* complete response, *PR* partial response, *SD* stable disease, *PD* progressive disease, *BCAA* branched-chain amino acid, *GI* gastrointestinal

### Association between baseline L3 SMI and survival

Thirty-one patients (30.4%; 14 men and 17 women) were classified into the low SMI group at baseline, whereas 71 patients (69.6%; 56 men and 15 women) were classified into the high SMI group (Table 2). Patient age (*P* = 0.001) and the proportion of women (*P* = 0.001) were significantly higher in the low SMI group than in the high SMI group. BMI was significantly higher in both men (*P* <  0.001) and women (*P* = 0.009) with high SMI than in those with low SMI. The most prevalent etiology of HCC was HCV infection in the low SMI group, and non-B non-C hepatitis in the high SMI group (*P* = 0.003). There were no significant differences in Child-Pugh classification, TNM stage, or BCAA supplementation between the low and high SMI groups.

**Table 2:** Comparison of demographic and clinical characteristics between low and high skeletal muscle index groups

Variables	Low SMI (No. = 31)	High SMI (No. = 71)	*P* value
Observation period, days, median [range]	740 [161–3278]	706 [153–2902]	0.708
Age, years, median [range]	74 [53–89]	66 [34–86]	0.001
Sex (men/women), *n*	14/17	56/15	0.001
BMI in men, kg/m^2^, median [range]	21.0 [16.2–25.8]	24.4 [19.7–33.8]	< 0.001
BMI in women, kg/m^2^, median [range]	22.0 [16.0–37.5]	23.9 [20.7–34.7]	0.009
Baseline L3 SMI in men, cm^2^/m^2^, median [range]	36.8 [31.5–40.7]	48.2 [42.2–64.4]	< 0.001
Baseline L3 SMI in women, cm^2^/m^2^, median [range]	34.4 [25.6–36.5]	41.7 [38.1–55.6]	< 0.001
Etiology (HBV/HCV/NBNC), *n*	1/23/7	10/27/34	0.003
ALT, U/L, median [range]	38 [13–123]	37 [9–243]	0.945
Total bilirubin, mg/dL, median [range]	0.8 [0.5–2.7]	1.0 [0.4–3.5]	0.107
Albumin, g/dL, median [range]	3.6 [2.1–4.7]	3.7 [2.0–4.9]	0.249
Platelet count, × 10^4^/μL, median [range]	11.6 [3.2–61.5]	12.1 [2.2–80.9]	0.636
Child-Pugh classification (A/B), *n*	20/11	48/23	0.821
AFP, ng/mL, median [range]	19.5 [2.1–633,900]	24.7 [1.0–285,973]	0.881
TNM stage (I + II/III + IV), *n*	9/22	24/47	0.818
Maximum tumor diameter, mm, median [range]	26 [13–150]	36 [9–198]	0.662
Number of tumors (solitary/multiple), *n*	9/22	20/51	1.000
Treatment sessions between CT exams (1/≥2, sessions), *n*	17/14	42/29	0.828
mRECIST assessment (non-PD/PD), *n*	16/15	37/34	1.000
BCAA supplementation (presence/absence), *n*	22/9	50/21	1.000

*BMI* body mass index, *L3 SMI* skeletal muscle index at third lumber vertebral level, *HBV* hepatitis B virus, *HCV* hepatitis C virus, *NBNC* non-B non-C, *ALT* alanine aminotransferase, *AFP* alpha-fetoprotein, *CT* computed tomography, *mRECIST* Modified Response Evaluation Criteria in Solid Tumors, *PD* progressive disease, *BCAA* branched-chain amino acid

**Fig. 1:**
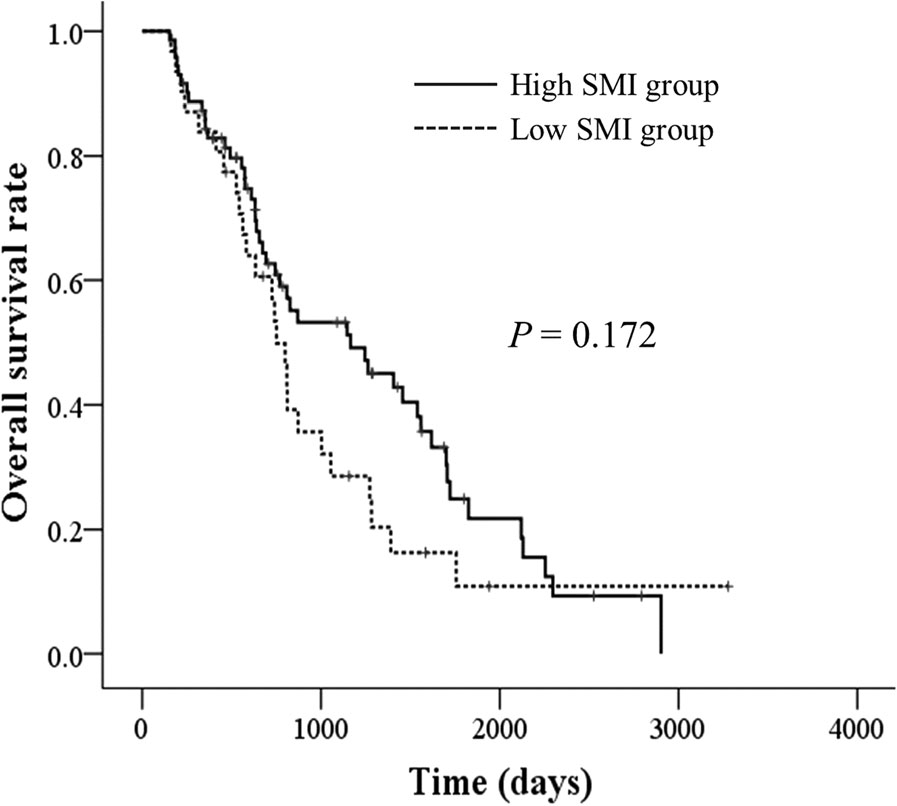
Overall survival rates in groups of patients with low and high skeletal muscle index (SMI) at the level of the third lumbar vertebra, as determined by cutoff values of 42 cm^2^/m^2^ for men and 38 cm^2^/m^2^ for women (*P* = 0.172). Survival rates were estimated with the Kaplan-Meier method and compared using log-rank tests

The OS rate tended to be higher in the high SMI group than in the low SMI group (*P* = 0.172), as shown in Fig. 1. Univariate analysis using a Cox proportional hazards model revealed no significant association between baseline L3 SMI and OS rate (HR, 1.405; 95% CI, 0.861–2.293; *P* = 0.174).

### Association between rate of change in skeletal muscle mass and survival

The cutoff value for ΔL3 SMI was determined to be − 4.6% over 6 months, according to the optimization method for classification [24, 25]. A sharp decrease in skeletal muscle mass less than the cutoff value was defined as skeletal muscle loss (SML); other decreases were defined as non-skeletal muscle loss (non-SML). Of the 102 study patients, 41 (40.2%; 30 men and 11 women) were classified into the SML group, and 61 (59.8%; 40 men and 21 women) were classified into the non-SML group (Table 3). Comparison of the demographic and clinical characteristics of patients in the SML and non-SML groups revealed that the observation period was significantly shorter and the serum albumin level significantly lower in the SML group than in the non-SML group. The SML group was also characterized by features of more advanced tumor progression, such as higher serum AFP level and larger tumor size.

**Table 3:** Comparison of demographic and clinical characteristics between skeletal muscle loss and non-skeletal muscle loss groups

Variables	SML (No. = 41)	Non-SML (No. = 61)	*P* value
Observation period, days, median [range]	632 [153–2902]	785 [184–3278]	0.021
Age, years, median [range]	67 [38–89]	72 [34–88]	0.231
Sex (men/women), *n*	30/11	40/21	0.515
BMI in men, kg/m^2^, median [range]	23.5 [17.6–32.7]	23.5 [16.2–33.8]	0.972
BMI in women, kg/m^2^, median [range]	22.8 [16.0–34.7]	23.2 [17.1–37.5]	0.858
ΔL3 SMI in men, %, median [range]	−10.1 [−34.2– −4.7]	−0.8 [−4.3–14.2]	< 0.001
ΔL3 SMI in women, %, median [range]	−8.3 [−25.2– −5.3]	1.4 [−4.5–13.0]	< 0.001
Etiology (HBV/HCV/NBNC), *n*	4/22/15	7/28/26	0.744
ALT, U/L, median [range]	47 (10–243)	37 (9–166)	0.188
Total bilirubin, mg/dL, median [range]	1.1 [0.4–2.4]	0.9 [0.5–3.5]	0.423
Albumin, g/dL, median [range]	3.6 [2.1–4.7]	3.7 [2.0–4.9]	0.026
Platelet count, ×10^4^/μL, median [range]	11.9 [3.4–80.9]	12.1 [2.2–27.1]	0.453
Child-Pugh classification (A/B), *n*	27/14	41/20	1.000
AFP, ng/mL, median [range]	54 [2.0–633,900]	14 [1.0–40,769]	0.009
TNM stage (I + II/III + IV), *n*	12/29	21/40	0.668
Maximum tumor diameter, mm, median [range]	50 [10–198]	28 [9–120]	0.009
Number of tumors (solitary/multiple), *n*	12/29	17/44	1.000
Treatment sessions between CT exams (1/≥ 2, sessions), *n*	24/17	35/26	1.000
mRECIST assessment (non-PD/PD), *n*	18/23	35/26	0.227
BCAA supplementation (presence/absence), *n*	30/11	42/19	0.665

*SML* skeletal muscle loss, *BMI* body mass index, *ΔL3 SMI* rate of change of skeletal muscle mass over 6 months, *HBV* hepatitis B virus, *HCV* hepatitis C virus, *NBNC* non-B non-C, *ALT* alanine aminotransferase, *AFP* alpha-fetoprotein, *CT* computed tomography, *mRECIST* Modified Response Evaluation Criteria in Solid Tumors, *PD* progressive disease, *BCAA* branched-chain amino acid

**Fig. 2:**
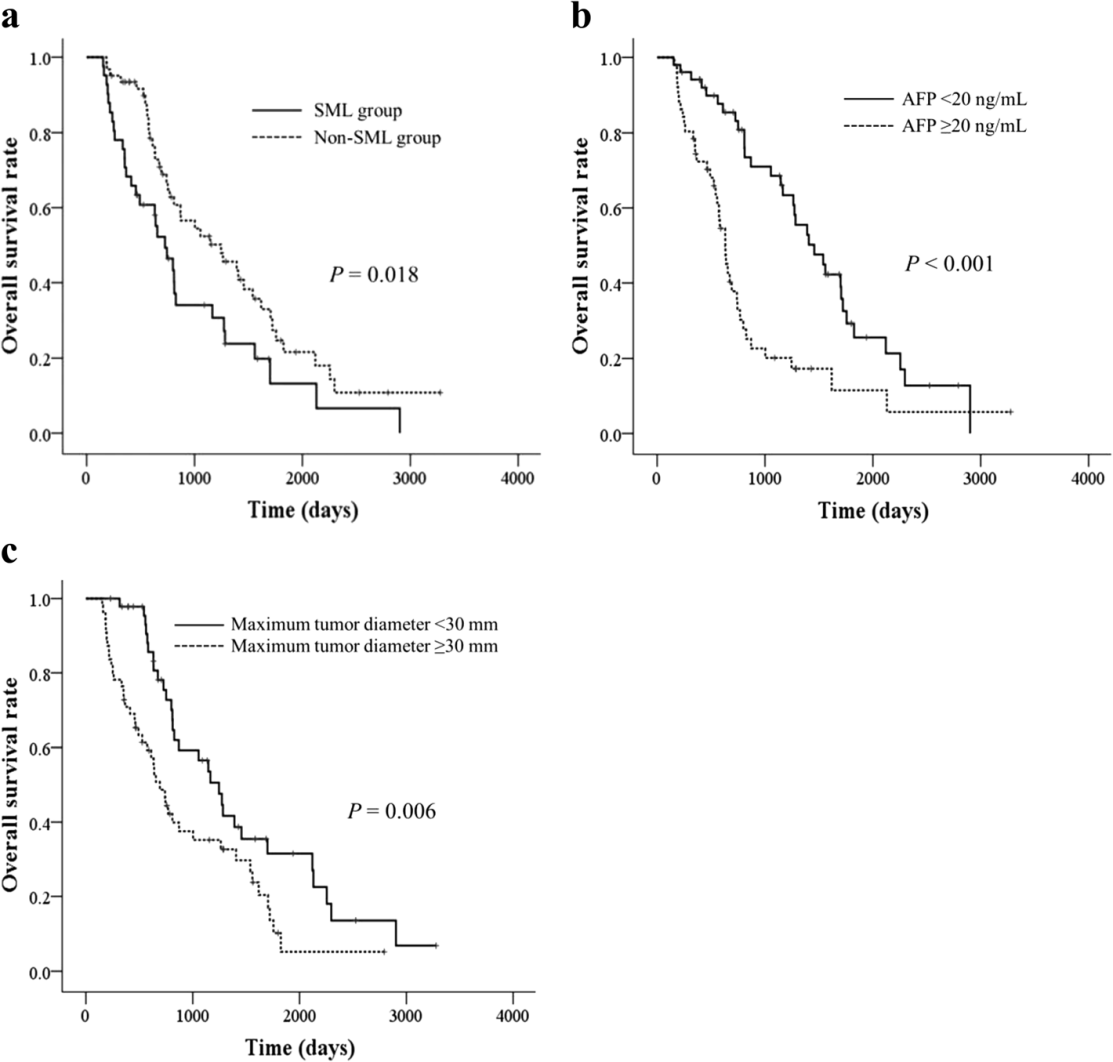
Overall survival rates in patients (**a**) with skeletal muscle loss (SML) or non-SML (*P* = 0.018), (**b**) with alpha-fetoprotein (AFP) < 20 ng/mL or ≥ 20 ng/mL (*P* <  0.001), and (**c**) with maximum tumor diameter <30 mm or ≥30 mm (*P* = 0.006). Survival rates were estimated with the Kaplan-Meier method and compared using log-rank tests

Univariate and multivariate analyses based on a Cox proportional hazards model were performed to identify independent predictors of survival (Table 4). Univariate analysis showed that SML; serum level of total bilirubin ≥1.5 mg/dL, albumin < 3.5 g/dL, and AFP ≥ 20 ng/mL; maximum tumor diameter ≥ 30 mm; and progressive disease in response to treatment were significantly associated with poor OS. Multivariate analysis revealed that SML (HR, 1.675; 95% CI, 1.031–2.721; *P* = 0.037), serum AFP ≥ 20 ng/mL (HR, 2.550; 95% CI, 1.440–4.515; *P* = 0.001), and maximum tumor diameter ≥ 30 mm (HR, 1.925; 95% CI, 1.166–3.179; *P* = 0.010) were independently predictive of poor OS (Fig. 2).

**Table 4:** Univariate and multivariate analysis using Cox proportional hazards model for overall survival

Variables	Univariate analysis		Multivariate analysis	
	HR (95% CI)	*P* value	HR (95% CI)	*P* value
Age, years, ≥70 vs. <70	0.857 (0.539–1.362)	0.513		
Sex, men vs. women	1.354 (0.814–2.255)	0.243		
BMI, kg/m^2^, ≥25.0 vs. <25.0	0.731 (0.444–1.204)	0.218		
Baseline L3 SMI, low SMI vs. high SMI	1.405 (0.861–2.293)	0.174		
ΔL3 SMI, SML vs. non-SML	1.750 (1.093–2.800)	0.020	1.675 (1.031–2.721)	0.037
ALT, U/L, ≥30 vs. <30	1.046 (0.650–1.682)	0.854		
Total bilirubin, mg/dL, ≥1.5 vs. <1.5	1.690 (1.002–2.851)	0.049	1.747 (0.897–3.403)	0.101
Albumin, g/dL, ≥3.5 vs. <3.5	0.595 (0.361–0.982)	0.042	0.697 (0.386–1.259)	0.231
Platelet count, ×10^4^/μL, ≥10.0 vs. <10.0	0.971 (0.607–1.552)	0.902		
Child-Pugh classification, A vs. B	0.638 (0.395–1.030)	0.066		
AFP, ng/mL, ≥20 vs. <20	2.618 (1.615–4.241)	< 0.001	2.550 (1.440–4.515)	0.001
TNM stage, I + II vs. III + IV	0.785 (0.474–1.300)	0.347		
Maximum tumor diameter, mm, ≥30 vs. <30	1.936 (1.195–3.135)	0.007	1.925 (1.166–3.179)	0.010
Number of tumors, solitary vs. multiple	0.961 (0.572–1.614)	0.880		
Treatment sessions between CT exams, sessions, 1 vs. ≥ 2	0.696 (0.433–1.119)	0.135		
mRECIST assessment, non-PD vs. PD	0.444 (0.277–0.712)	0.001	0.653 (0.392–1.088)	0.102
BCAA supplementation, presence vs. absence	1.614 (0.898–2.900)	0.110		

*HR* hazard ratio, *CI* confidence interval, *SML* skeletal muscle loss, *BMI* body mass index, *L3 SMI* skeletal muscle index at third lumber vertebral level, *ΔL3 SMI* rate of change of skeletal muscle mass over 6 months, *SML* skeletal muscle loss, *ALT* alanine aminotransferase, *AFP* alpha-fetoprotein, *CT* computed tomography, *mRECIST* Modified Response Evaluation Criteria in Solid Tumors, *PD* progressive disease, *BCAA* branched-chain amino acid

## Discussion

This retrospective study evaluated whether baseline skeletal muscle mass and its rate of change over 6 months were prognostic factors for OS in patients with HCC treated with transcatheter intra-arterial therapies. To our knowledge, this study is the first to show that the rate of change in skeletal muscle mass over 6 months was an independent indicator of clinical outcomes in these patients.

Prognostic factors that have been previously identified in patients with HCC treated with TACE or TAI include hepatic functional reserve; tumor-specific factors, such as number, diameter, vascular invasion, and distribution in the liver; and serum AFP concentrations [8–11]. The present study additionally revealed that the 6-month rate of change in skeletal muscle mass was predictive of survival in these patients. In previous studies, an association between change in skeletal muscle mass during chemotherapy and prognosis has been demonstrated in patients with various malignancies other than HCC [28–32]; these findings indicate that the same association may be applied to predict outcomes in patients with HCC undergoing chemotherapeutic treatment. Because CT examinations are usually repeated at intervals shorter than 6 months as part of the clinical workup for patients with HCC, it is quite feasible to practically apply our method of quantification of skeletal muscle mass.

Skeletal muscle mass has been reported to decline by approximately 1.4% per year owing to physiological aging [33]. In patients with cirrhosis, skeletal muscle is lost more rapidly, declining by about 2.2% per year [34]. The present study found that patients with HCC treated with transcatheter intra-arterial therapies showed drastic decreases in skeletal muscle mass, with a median rate of − 3.5% over 6 months. Furthermore, SML < − 4.6% over 6 months was significantly associated with poorer OS. These findings suggest that a rapid loss of skeletal muscle mass induced by deterioration of hepatic function is associated with poor clinical outcomes in patients with chronic liver disease.

In the current study, skeletal muscle mass at baseline was not predictive of clinical outcomes, in contrast to the findings of previous studies in which patients with HCC were treated with modalities other than transcatheter intra-arterial therapies [14–20]. This discrepancy may be the result of differences in hepatic functional reserve or tumor progression among the enrolled patients. Guidelines for the treatment of HCC recommend radical therapies in patients with preserved liver function and limited tumor progression [22], who often have good nutritional status and preserved skeletal muscle mass. In contrast, most patients who undergo transcatheter intra-arterial therapies have more severely impaired liver function, or tumors in more advanced stages. These adverse conditions may have a considerable impact on outcomes, irrespective of skeletal muscle mass at baseline.

Although we did not assess the mechanisms responsible for the rapid loss of skeletal muscle in the patients with HCC in the present study, the phenomenon may be associated with protein-energy malnutrition (PEM). PEM is frequently observed in patients with liver disease, and facilitates catabolism and loss of skeletal muscle mass [35–37]. In addition, patients with impaired liver function are often deficient in vitamin D or carnitine, which might cause skeletal muscle atrophy [38, 39]. Most of the patients in our study had severely deteriorated liver function and HCC in advanced stages; these detrimental conditions may have led to rapid depletion of skeletal muscle.

Although prevention of SML has not been shown to improve the prognosis of patients with HCC, dietary supplementation with BCAAs in patients with cirrhosis or HCC was found to preserve liver function and increase muscle protein synthesis by activating the mammalian target of rapamycin signaling [40, 41]. Vitamin D supplementation may also inhibit tumor progression [42–44] and increase muscle mass and strength [45–47]. Furthermore, supplementation with L-carnitine may prevent skeletal muscle atrophy by activating the insulin-like growth factor-1/AKT/p70S6 kinase pathway [48]. Physical exercise also has beneficial effects on muscles through attenuation of signaling pathways associated with skeletal muscle proteolysis in cachectic conditions and promotion of protein synthesis via anti-inflammatory and anti-oxidative effects [49, 50]. However, a recent study showed that exercise alone could not prevent a reduction in skeletal muscle mass, but that BCAA supplementation minimized skeletal muscle depletion in patients with HCC [51]. It will be necessary to investigate whether the conservation of skeletal muscle with a combination of nutritional support and exercise could improve the prognosis of patients with HCC, with the goal of developing a systematic strategy for the preservation of skeletal muscle in these patients.

The present study had several limitations. First, the study involved a relatively small number of patients who were treated at a single institution. Second, our study was retrospective in design, and we could not evaluate skeletal muscle strength and function. The assessment of not only skeletal muscle mass but also strength or function is needed to diagnose sarcopenia, according to guidelines [21]. Prospective studies assessing skeletal muscles comprehensively among larger numbers of patients with HCC are required to confirm our results.

## Conclusions

In this study, we found that the rate of change in skeletal muscle mass over 6 months was an independent prognostic factor in patients with HCC treated with transcatheter intra-arterial therapies. In contrast, SML at baseline was not prognostic of clinical outcomes in these patients. Further study is required to reveal whether prevention of skeletal muscle depletion might contribute to improvement of clinical outcomes in patients with HCC, as a new therapeutic strategy.

## Abbreviations

AFP: Alpha-fetoprotein
ALT: Alanine aminotransferase
BCAA: Branched-chain amino acid
BMI: Body mass index
CI: Confidence interval
CT: Computed tomography
HBV: Hepatitis B virus
HCC: Hepatocellular carcinoma
HCV: Hepatitis C virus
HR: Hazard ratio
L3 SMI: Skeletal muscle index at the third lumbar vertebral level
mRECIST: Modified Response Evaluation Criteria in Solid Tumors
MRI: Magnetic resonance imaging
OS: Overall survival
PEM: Protein-energy malnutrition
RFA: Radiofrequency ablation
SML: Skeletal muscle loss
TACE: Transcatheter arterial chemoembolization
TAI: Transcatheter arterial infusion chemotherapy
